# Failure of a repeat course of cyclooxygenase inhibitor to close a PDA is a risk factor for developing chronic lung disease in ELBW infants

**DOI:** 10.1186/1471-2431-12-10

**Published:** 2012-01-27

**Authors:** Lynda Adrouche-Amrani, Robert S Green, Karen M Gluck, Jing Lin

**Affiliations:** 1 Mount Sinai Kravis Children's Hospital and Department of Pediatrics, Mount Sinai School of Medicine, One Gustave L. Levy Place, New York, NY 10029-6574, USA

## Abstract

**Background:**

The optimal treatment regimen or protocol for managing a persistent patent ductus arteriosus (PDA) in extremely low birth weight (ELBW) infants has not been well established. This study was aimed at evaluating the failure rate of a cyclooxygenase (COX) inhibitor (COI) for PDA closure and to determine the incidence of a PDA requiring ligation in ELBW infants. We examined the clinical characteristics and risk factors that may predict the clinical consequences of failure of PDA closure by COI.

**Methods:**

Medical information on 138 infants with birth weight (BW) < 1000 gm who survived for > 48 hours was retrieved. Clinical characteristics and outcomes of patients whose PDAs closed with COI were compared with those who did not close.

**Results:**

Of the 138 patients, 112 survived to discharge. Eighty (71.4%) of those who survived received 1-3 courses of COI treatment for a symptomatic PDA. A total of 32 (40%) failed COI treatment and underwent PDA ligation. Multivariable logistic regression analysis suggests that the observed differences in the outcomes in infants with or without symptomatic PDA can be explained by the babies with symptomatic PDA being more immature and sicker. No significant difference was seen in the incidence of chronic lung disease (CLD) in infants whose PDA was treated medically versus those who failed medical treatment and then underwent ligation. However, after adjusting for disease severity and other known risk factors, the odds ratio of developing CLD for surviving babies with a persistent PDA compared to those whose PDA was successfully closed with 1-2 courses of COI is 3.24 (1.07-9.81; p = 0.038).

**Conclusions:**

When successfully treated, PDA in ELBW infants did not contribute significantly to the adverse outcomes such as CLD, retinopathy of prematurity (ROP) and age at discharge. This suggests that it is beneficial for a hemodynamically significant PDA to be closed. The failure of a repeat course of COI to close a PDA is a major risk factor for developing CLD in ELBW infants.

## Background

Spontaneous closure of the ductus arteriosus (DA) usually occurs within hours to days after birth in term infants. However, the incidence of failure of DA closure in premature infants ranges from 10% to 60% depending on the gestational age, birth weight and diagnostic criteria used [1,2]. In extremely low birth weight (ELBW) infants (birth weight less than 1000 grams), only 34% have a spontaneous permanent closure of the DA [3]. A significant left-to-right shunt through the PDA may increase morbidity and therefore contribute to mortality in premature infants. The increased pulmonary blood flow due to a left to right shunt through a PDA can lead to deterioration of the respiratory status of premature infants and may contribute to the development of bronchopulmonary dysplasia (BPD) or chronic lung disease (CLD) [4]. A patent ductus arteriosus (PDA) with a significant left-to-right shunt may also increase the risk for renal insufficiency, reduced brain perfusion [5] and possible necrotizing enterocolitis (NEC) [6].

Although indomethacin, a cyclooxygenase inhibitor (COI), is very effective in closing the DA of preterm infants [7,8], its efficacy is limited in very immature infants [9]. In ELBW infants, the success rate of DA closure after the first course of indomethacin has been reported to be between 20 and 40% [9,10]. When a PDA fails to close after medical management or when treatment with COI is contraindicated, surgical ligation is an alternative. Currently, most neonatologists use repeat courses of indomethacin or ibuprofen in an attempt to close the PDA before committing the patient to surgical ligation [6,11]. Recent studies have suggested an increased risk of CLD, ROP and/or neurosensory impairment may be associated with PDA ligation [12,13]. However, it is unclear whether the clinical condition of the baby requiring surgical ligation or the procedure itself contributes to an adverse outcome [2]. Therefore, the optimal treatment regimen for a persistent PDA in ELBW infants has not been established. The aim of the current study was to evaluate the clinical course of ELBW infants with a PDA treated with COI, the rate of failure of COI for PDA closure and the incidence of PDA requiring surgical ligation. We compared the clinical characteristics and outcomes of babies whose PDA were successfully closed by COI with those whose DA remained patent after COI treatment and determined if any of these factors contributed to the clinical outcomes of the ELBW infants.

## Methods

We performed a retrospective data analysis. Medical information of infants with a birth weight (BW) < 1000 gm born at the Mount Sinai Medical Center in New York between August 1, 2004 and July 31, 2009 was retrieved from our perinatal data base and medical records. The study was approved by the Program for the Protection of Human Subjects of the Mount Sinai Medical Center. It was our practice during that period to: 1) perform echocardiography only when a PDA was suspected due to the presence of a murmur, wide pulse pressure, or deterioration of pulmonary status possibly secondary to left to right shunting; 2) treat a PDA with COI in infants who were diagnosed with a hemodynamically significant PDA as judged by our attending neonatologists and/or confirmed by echocardiography; 3) use indomethacin as the preferred choice, but use ibuprofen for some babies with decreased urine output; 4) not use indomethacin for prophylactic ductal closure in infants less than 48 hours of age; 5) give indomethacin 0.2 mg/kg every 12 hours for 3 doses or ibuprofen 10 mg/kg followed by 5 mg/kg daily for 2 more doses as one course; 6) obtain echocardiagraphy confirmation of a moderate to large PDA with significant left to right shunt before surgical ligation; 7) give a third course of COI when PDA ligation could not be done expeditiously due to scheduling issues. We collected clinical data and determined the incidence of spontaneous closure of the DA, the failure rate of 1-3 courses of COI for PDA, the incidence of PDA treated with surgical ligation, and the clinical outcomes of these infants. Successful PDA closure was defined as disappearance of the PDA murmur and/or echocardiogram evidence of PDA closure. Echocardiographic evidence of trivial flow through DA and no further treatment needed was considered to have closed PDA.

The clinical data collected included gestational age, birth weight, gender, Apgar scores, admission temperature, first arterial blood gas, surfactant treatment, duration of intubation for mechanical ventilation, total fluid administered during the first three days of life, and the age when an infant received the first course of COI. The urine output, serum sodium concentration, body weight and total fluid status before, during and after the first course of COI were also recorded. The disease severity was quantified by the oxygenation index (OI) and the five-item clinical risk index for babies (CRIB) II score as described [14,15]. The clinical outcome measurements include the age at extubation, the age at full enteral feeds (defined as baby receiving no intravenous fluid and > 120 ml/k.day of enteral feeds), the incidence of severe (grade 3 or grade 4) intraventricular hemorrhage (IVH), CLD (defined as supplemental oxygen requirement at 36 weeks postmenstrual age), ROP, necrotizing enterocolitis (NEC) with Bell stage II or higher, blood culture proven bacteremia, and the age at discharge.

Data analysis was conducted with PASW Statistics 18.0 (SPSS Inc.). Continuous variables between groups were compared using the T-test for independent samples for normally distributed data and the Mann-Whitney test for non-normally distributed data. Proportions between groups were compared using the Chi-square test. For dichotomous outcomes, odds ratios adjusted for confounding and effect modifiers were determined using logistic regression as indicated in the text. For continuous outcome varibles, multivariable regression analysis was used to determine the effect of a PDA and its treatment as indicated in the text. The data are presented as mean (standard error of the mean) and P < 0.05 is considered significant.

## Results

A total of 189 infants with a BW < 1000 gm were born at the Mount Sinai Hospital between August 1, 2004 and July 31, 2009. Of those patients, 14 patients with major congenital anomalies and 37 who died at age < 48 hours of life were excluded from the analysis.

Data from the remaining 138 patients were reviewed. Among those 138 patients, 26 died before discharge. Seven of those 26 died at age ≤ 7 days of life. The causes of death for those 26 patients were mainly respiratory failure due to extremely immature lungs (13/26). Other causes of death include NEC or sepsis (9/26), severe IVH (2/26), and twin to twin transfusion (2/26). Of the 112 babies who survived to discharge, 32 (28.6%) did not develop a hemodynamically significant PDA (the DA closed spontaneously). The remaining 80 patients (71.4%) were diagnosed with a hemodynamically significant PDA and received at least one course of COI. Among the 80 patients who received COI treatment for a PDA, 26 (32.5%) infants closed their PDA after one course of COI. One infant had her PDA ligated after the failure of one course of COI to achieve ductal closure due to significant side effects of COI usage. The remaining 53 patients received a second course of COI and 16 of these patients (30.2%) closed their PDA after the second course of COI. Therefore, there were a total of 37 of 80 patients (46.2%) whose DA remained clinically significant after 2 courses of COI treatment. PDAs were ligated in 22 out of these 37 patients after the second course and the other 15 babies received a third course of COI. Among the 15 infants who received a third course, 6 (40%) closed their PDA and the remaining 9 infants underwent PDA ligation. Therefore, a total of 32/80 (40%) underwent surgical ligation of the PDA. Most of the ligations were performed in the second or third week of life.

Detailed analysis was done on those 112 patients who survived to discharge. The demographics of infants with vs. those without hemodynamically significant PDAs are shown in Table 1. The babies with hemodynamically significant PDAs were less mature, had lower birth weights, higher CRIB II scores, higher OI, and received more fluid during the first 3 days of life. The overall outcomes for babies with hemodynamically significant PDAs were worse, as evidenced by an older age at extubation, reaching full enteral feeds later, and having a longer duration of hospitalization. Furthermore, the babies with hemodynamically significant PDAs had a higher incidence of CLD and ROP.

**Table 1:** Demographics of the ELBW infants who survived to discharge

	No PDA (n = 32)	PDA (n = 80)	P
BW, gm	815 (20)	753 (14)	0.015
GA, wks	27.3 (0.4)	25.2 (0.1)	< 0.001
Male Gender (%)	14 (44%)	38 (48%)	ns
CRIB II score	9.9 (0.4)	12.4 (0.2)	< 0.001
OI	3.1 (0.5)	4.2 (0.3)	0.033
Surfactant use	23 (71.9%)	77 (96.3%)	0.001
Intubated > 2 days	18 (56.3%)	71 (88.8%)	< 0.001
Fluid 1^st ^3 days, ml/k/d	102 (3)	113 (2)	0.045
CLD	10 (31.3%)	42 (52.5%)	0.042
NEC	3 (9.4%)	8 (10.0%)	ns
IVH III-IV	0 (0.0%)	6 (7.5%)	ns
ROP	6 (18.8%)	51 (63.8%)	< 0.001
Age at extubation, days	8 (3)	29 (4)	< 0.001
Age at full feeds, days	21 (1)	33 (2)	< 0.001
Age at discharge, days	83 (5)	105 (6)	0.041

In order to ascertain whether these adverse outcomes in the babies with hemodynamically significant PDA might be attributable to the pre-existing risk factors of lower birth weight, lower gestational age, higher CRIB II scores, and higher OIs in these infants, we performed a multivariable logistic regression analysis. Since both birth weight and gestational age are included in the CRIB II score, logistic regression with CLD as the clinical outcome adjusted for CRIB II and OI was performed. This analysis reveals an adjusted odds ratio for CLD in those with vs. those without PDA of 1.10 (95% confidence interval [CI]: 0.37 - 3.30, P = 0.867). Similarly, the adjusted odds ratio for ROP in those with vs. those without symptomatic PDA is 3.20 (95% CI: 0.93 - 11.10, P = 0.065). These data suggest that the observed difference in CLD and ROP in babies with hemodynamically significant PDA vs. those without can be explained by the babies with hemodynamically significant PDA being less mature, having a lower birth weight, and being sicker as indicated by higher CRIB II scores and OI. Furthermore, multivariable regression analysis shows that a hemodynamically significant PDA did not add significantly to either CRIB II score or gestational age alone as a predictor of age at extubation or age at discharge. However, a multivariable regression analysis shows that the addition of a hemodynamically significant PDA to either CRIB II (R^2 ^increases from 0.176 to 0.234 by addition of PDA to the model, p = 0.006) or gestational age (R^2 ^increases from 0.195 to 0.232 by addition of PDA to the model, p = 0.025) improves a regression model for predicting age at full feeds. Presence of a hemodynamically significant PDA prolongs the time to full feeds by 8.0 ± 2.8 days in the multivariable model with CRIB II score and by 6.8 ± 3.0 days in the model with gestational age.

Among the 80 patients who received COI treatment for PDA, 26 (32.5%) infants closed their PDA after one course of COI. The comparisons of the infants whose DA closed with 1^st ^course vs. who did not close with 1^st ^course are presented in Table 2. As shown in the Table 2, other than the babies whose PDA was closed with the first course of COI being slightly more mature than those whose PDA did not close, none of the factors that we examined distinguished infants who responded to one course COI vs. those who did not.

**Table 2:** Comparisons of the ELBW infants whose DA closed with 1^st ^course of COI vs. who did not close with 1^st ^course

	Closed (n = 26)	Not Closed (n = 54)	P
BW, gm	764 (23)	749 (17)	ns
GA, wks	25.6 (0.2)	25.0 (0.1)	0.014
Male Gender (%)	14 (54%)	24 (44%)	ns
CRIB II score	12.0 (0.4)	12.6 (0.3)	ns
OI	3.4 (0.3)	4.5 (0.3)	ns
Age 1^st ^Dose, days	5.5 (0.8)	6.8 (1.4)	ns
Fluid 1^st ^3 days, ml/k/d	114 (5)	113 (2)	ns
UOP before Rx, ml/k/h	3.7 (0.2)	3.9 (0.2)	ns
UOP during Rx, ml/k/h	3.3 (0.3)	3.2 (0.2)	ns
UOP after Rx, ml/k/h	3.3 (0.3)	3.2 (0.2)	ns

The baseline characteristics of infants whose PDA closed with either one course or two courses of COI vs. those whose DA remained patent after 2nd course of COI are presented in Table 3. The babies whose PDA closed with either one or two courses of COI were slightly more mature than those who had persistent hemodynamically significant PDA but were otherwise not different (we excluded the baby whose PDA was ligated after the first course of COI). As is shown in Table 4, the incidence of CLD in infants whose DA remained patent after two courses of COI was almost twice that of babies whose DA closed with either one or two courses of COI. Furthermore, logistic regression analysis shows that, when compared to those whose PDA was successfully closed with the first or second course of COI, after adjusting for CRIB II score, OI, intubation for more than 2 days, and culture proven later onset bacteremia, the odds ratio of having CLD for surviving babies with persistent hemodynamically significant PDA is 3.24 (95% CI: 1.07 - 9.81, p = 0.038). Adding PDA ligation as a potential confounder did not improve the logistic model. These data demonstrate that failure of closure of PDA after 2 courses of COI is a significant risk factor for development of CLD in ELBW infants.

**Table 3:** Baseline characteristic of the ELBW infants whose DA was closed after 2nd course COI vs. those whose DA remained patent after 2nd course

	Closed (n = 42)	Not Closed (n = 37)	P
BW, gm	777 (17)	724 (21)	ns
GA, wks	25.5 (0.2)	24.9 (0.2)	0.007
Male Gender (%)	23 (55%)	15 (41%)	ns
CRIB II score	12.0 (0.3)	12.9 (0.4)	ns
OI	3.7 (0.3)	4.7 (0.5)	ns
Intubated > 2 days	36 (85.7%)	34 (91.9%)	ns
Age 1^st ^Dose, days	5.6 (0.6)	7.3 (2.0)	ns

**Table 4:** Outcomes of the ELBW infants whose DA was closed after 2nd course COI vs. those whose DA remained patent after 2nd course

	Closed (n = 42)	Not Closed (n = 37)	P
CLD	16 (38.1%)	25 (67.6%)	0.013
NEC	5 (11.9%)	3 (8.1%)	ns
IVH III-IV	3 (7.1%)	3 (8.1%)	ns
ROP	27 (64.3%)	24 (64.9%)	ns
Age at extubation, days	27 (6)	33 (5)	ns
Age at full feeds, days	33 (2)	34 (2)	ns
Age at discharge, days	101 (4)	109 (13)	ns

Table 5 shows a comparison of infants with PDA which closed with one (n = 26), two (n = 16), or three (n = 6) courses of COI vs. those who underwent surgical ligation after failure of one (n = 1), two (n = 22), or three (n = 9) courses of COI. Other than the fact that babies whose DAs were ligated received more courses of COI, neither the risk factors nor the outcomes we examined distinguished the babies who underwent ligation from those who did not.

**Table 5:** Comparisons of ELBW infants with PDA closed by COI treatment vs. those with PDA who failed medical treatment and underwent surgical ligation

	COI Only (n = 48)	COI & Ligation (n = 32)	P
BW, gm	772 (16)	726 (24)	ns
GA, wks	25.4 (0.2)	24.9 (0.2)	ns
Male Gender (%)	26 (54%)	12 (38%)	ns
CRIB II score	12.1 (0.3)	12.9 (0.4)	ns
OI	3.9 (0.3)	4.5 (0.5)	ns
COI courses	1.6 (0.1)	2.3 (0.1)	< 0.001
Age 1^st ^Dose, days	5.6 (0.6)	7.5 (2.3)	ns
CLD	21 (43.8%)	21 (65.6%)	ns
NEC	6 (12.5%)	2 (6.3%)	ns
IVH III-IV	4 (8.3%)	2 (6.3%)	ns
ROP	29 (60.4%)	22 (68.8%)	ns
Age at extubation, days	27 (5)	33 (5)	ns
Age at full feeds, days	33 (2)	34 (2)	ns
Age at discharge, days	102 (4)	110 (14)	ns

## Discussion

In this retrospective data analysis study, we found that less than one third of the ELBW infants who survived to discharge closed their DA spontaneously. The babies who had spontaneous closure of their DA were more mature, had a higher birth weight, and were less ill as indicated by lower CRIB II scores and lower OI. As compared to those who had a symptomatic PDA requiring treatment, the overall outcomes for the babies who closed their DA spontaneously were better, as demonstrated by a younger age at extubation, reaching full enteral feeds earlier, and having a shorter duration of hospitalization, and with lower incidences of CLD and ROP. These data are well known and consistent with numerous reports in the literature [3,16-18]. However, our analysis using multivariable logistic regression suggests that the observed difference in outcomes such as CLD and ROP in ELBW infants with a symptomatic PDA vs. those without can be explained by the babies with a symptomatic PDA being less mature and sicker (by CRIB II and OI). Furthermore, regression analysis shows that a hemodynamically significant PDA as managed in our NICU does not add significantly to either CRIB II score or gestational age alone as predictors of age at extubation or age at discharge.

Recent papers have suggested that there is little evidence of benefit of closing a PDA in extremely preterm infant and that potential side effects and complications related to COI and surgery are significant [6,19]. However, in a recent study from Western Australia, where surgical ligation was not practical, persistent PDA when left untreated was associated with much higher mortality, even after adjustment was made for initial disease severity, gestational age and other perinatal factors [20]. In addition to their being a well accepted association between CLD and PDA, our data also suggest that it is beneficial for a hemodynamically significant PDA in ELBW infants to be closed. This evidence supports the current practice of closing all hemodynamically significant PDAs in ELBW infants.

The presence of a hemodynamically significant PDA prolongs the time to full feedings by approximately one week in the multivariable model with either CRIB II score or gestational age. This is not surprising since it was our practice to routinely withhold feedings in babies whom we treated for PDA. The practice of withholding feedings or not initiating enteral feeds in babies who are receiving COI was based mainly on the concern that use of indomethacin may increase the risk for spontaneous intestinal perforation [21]. Indeed, by using multivariate regression analysis and two different derivations with a national dataset, Attridge et al. found significant associations between early use of indomethacin and spontaneous intestinal perforation [22]. However, they did not find an association with indomethacin when it was given after day of life four [22]. Therefore, withholding enteral feedings may not be necessary in extremely premature infants who are receiving COI treatment for PDA after day of life four.

In our NICU, COI treatment was initiated relatively late as compared to other published studies, and this may explain our relatively higher failure rate of COI treatment for PDA. We do not routinely perform screening echocardiograms in all ELBW infants in our NICU. It has been shown that a conservative approach for PDA management was associated with decreased rates of surgical ligation without significantly increased morbidity in extremely premature infants [23]. In the current study, surgical ligation was reserved for PDA closure when treatment with COI failed or was contraindicated. No indomethacin was given within 48 hours of age as prophylaxis and most surgical ligation happened between 2-3 weeks of life. Complications of surgical ligation were not observed in our series. Recently, a few studies have reported that surgical ligation is a risk factor associated with CLD [13,24]. Whether this association is related to surgical ligation, the PDA itself, or just extreme prematurity has been a matter of debate among neonatologists. Although in our study no statistically significant difference was seen in the incidence of CLD in infants whose PDA responded to COI treatment versus those who failed and then underwent surgical ligation, our numbers are quite small. Interestingly, the incidence of CLD in infants whose DA remained patent after 2 courses of COI was almost twice that of babies whose DA closed with either the first or second course of COI. After adjusting for CRIB II score, OI, intubation for more than 2 days, and culture proven bacteremia, the odds ratio of having CLD in surviving babies with persistently hemodynamically significant PDA is still significantly higher when compared to those whose PDA was successfully closed with one or two courses of COI. This may suggest that the failure of closure of a PDA with a second course of COI rather than surgical ligation is a significant risk factor for developing CLD in ELBW infants.

Adding PDA ligation as a factor did not improve the logistic model for an adjusted odd ratio of having CLD in ELBW infants who did not respond to a 2nd course of COI. This may suggest that some intrinsic factors which cause the DA to fail to respond COI treatment may contribute to the pathogenesis of CLD. However, our study is limited due to its retrospective nature. Furthermore, due to the small number of patients in our study and the arbitrary nature of the decisions to pursue surgical ligation vs. a third course of COI after failure of a 2nd course of COI, we cannot comment on the potential role of the third course of COI for closure of PDA and the role of surgical ligation in the development of CLD. Nevertheless, it may be beneficial to indentify potential clinical factors that predict the failure of COI treatment for PDA to guide the clinical management of PDA in ELBW infants. Unfortunately, we, as well as others [25,26], are unable to identify any clinical factors which can be reliably used to predict which specific infant will fail to close a hemodynamically significant PDA with COI treatment. More recently, it was demonstrated that using echocardiagraphy to direct the use of COI may lead to fewer doses of COI for PDA closure in premature infants [27]. This approach requires the availability of frequent echocardiographic evaluation, which may be problematic in many neonatal intensive care units.

## Conclusions

In conclusion, 71.4% of ELBW infants who survived to discharge were diagnosed with hemodynamically significant PDA and received treatment. When treated, PDA in ELBW infants did not contribute significantly to adverse outcomes such as CLD, ROP and age at discharge, suggesting that it is beneficial for a hemodynamically significant PDA to be closed. The incidence of CLD in infants whose DA remained patent after a 2nd course of COI was almost twice that of those whose DA closed with either one or two courses of COI. The adjusted odds of having CLD for surviving babies with persistent hemodynamically significant PDA is greater than three times that of babies whose PDA was successfully closed with either one or 2 courses of COI. Adding PDA ligation as a factor did not improve the logistic model for adjusted odd ratio of having CLD in those infants. This may suggest that persistent patency of the DA after two courses of COI rather than PDA ligation is a significant risk factor for developing CLD in ELBW infants. Due to small numbers of infants who responded to a third course of COI and the lack of obvious benefit, we can not draw any conclusions regarding to the use of third course COI in ELBW infants with hemodynamically significant PDA who failed the 2nd course of COI. The identification of reliable factors or biomarkers to guide the use of COI [28] or surgery may ultimately improve the outcomes of ELBW infants with hemodynamically significant PDA and should be the direction of future studies.

## List of abbreviations used

**BPD**: Bronchopulmonary Dysplasia; **CI**: Confidence Interval; **CLD**: Chronic Lung Disease; **COI**: Cyclooxygenase Inhibitor; **CRIB**: Clinical Risk Index for Babies; **DA**: Ductus Arteriosus; **ELBW**: Extremely Low Birth Weight; **OI**: Oxygenation Index; **PDA**: Patent Ductus Arteriosus; **ROP**: Retinopathy of Prematurity; **UOP**: Urine Output.

## Competing interests

The authors declare that they have no competing interests.

## Authors' contributions

LA carried out the data collection and drafted the manuscript. KG participated in the data collection. RG participated in the design of the study, data collection, and performed the statistical analysis. JL conceived of the study, and participated in its design and coordination and helped to draft the manuscript. All authors read and approved the final manuscript.

## Pre-publication history

The pre-publication history for this paper can be accessed here:

http://www.biomedcentral.com/1471-2431/12/10/prepub
